# Harnessing γδ T Cells against Human Gynecologic Cancers

**DOI:** 10.3390/life14030325

**Published:** 2024-02-29

**Authors:** Jose R. Conejo-Garcia, Carmen M. Anadon, Luis U. Lopez-Bailon, Ricardo A. Chaurio

**Affiliations:** 1 Department of Integrative Immunobiology, Duke School of Medicine, Durham, NC 27710, USAluis.lopez-bailon@duke.edu (L.U.L.-B.); ricardo.chauriogonzalez@duke.edu (R.A.C.); 2 Duke Cancer Institute, Duke School of Medicine, 2128 MSRBIII, 3 Genome Ct, Durham, NC 27707, USA

**Keywords:** T cell, γδ T cell, cancer immunotherapy, chimeric antigen receptor, tumor immunology, butyrophilin

## Abstract

Immuno-oncology has traditionally focused on conventional MHC-restricted αβ T cells. Yet, unconventional γδ T cells, which kill tumor cells in an MHC-unrestricted manner, display characteristics of effector activity and stemness without exhaustion and are nearly universally observed in human gynecologic malignancies, correlating with improved outcomes. These cells do not have a clear counterpart in mice but are also found in the healthy female reproductive tract. Interventions that modulate their in vivo activity, or cellular therapies utilizing γδ T cells as an allogeneic, “off-the-shelf” platform (e.g., for chimeric antigen receptor expression) hold significant potential against challenging tumors like ovarian cancer, which has been stubbornly resistant to the immune checkpoint inhibitors that change the landscape of other human tumors. Here, we discuss recent discoveries on the specific populations of γδ T cells that infiltrate human gynecologic cancers, their anti-tumor activity, and the prospect of redirecting their effector function against tumor cells to develop a new generation of immunotherapies that extends beyond the traditional αβ T cell-centric view of the field.

## 1. Introduction

While recent clinical trials have underscored the potential of an existing immune checkpoint blockade to target DNA mismatch repair-deficient endometrial cancers, most patients with gynecological malignancies—particularly ovarian cancer—still require more effective immunotherapies. Immuno-oncology has traditionally focused on understanding and targeting the role of αβ T cells in anti-tumor immunity, whereas the contribution of unconventional T cells, and particularly γδ T cells, remains poorly understood.

γδ T cells are a subset of T lymphocytes that express a T-cell receptor (TCR) containing γ and δ chains. γδ T cells are primarily CD4^−^CD8^−^ lymphocytes and represent up to 5% of total CD3^+^ T cells in circulation [1]; however, specific subsets of γδ T cells are enriched in various healthy tissues, including mucosal locations, with proportions ranging from 1% to 10% of the total human CD3^+^ T-cell population [2]. Extensive infiltration of human tumors by γδ T cells, including in ovarian cancer [3], has been reported in the range of 3–15% of total tumor-infiltrating T lymphocytes (TILs) [3,4,5,6,7,8]. These γδ T lymphocytes produce a variety of cytokines, have the capacity to cross-present antigens, and can re-direct antibodies against target cells through antibody-dependent cellular cytotoxicity. Unlike their conventional αβ T cell counterparts, which recognize peptides presented by major histocompatibility complex (MHC) molecules, γδ T cells possess a broader and incompletely understood recognition repertoire. Various γδ T-cell receptors (TCRs) can identify diverse antigens and butyrophilins (BTN) or BTN dimers, while their cytotoxic activity is primarily mediated through NKG2D signaling or other natural killer cell receptors (NKRs), prompting the release of cytotoxic molecules such as perforin and granzymes.

In recent years, γδ T cells have gained attention in immuno-oncology because of their association with spontaneous anti-tumor immunity against multiple human cancers, including ovarian and endometrial cancers, and their potential as an allogeneic, “off-the-shelf” platform for cellular therapies. Antibodies targeting butyrophilins and drugs promoting the accumulation of phosphometabolites (e.g., zoledronate) have shown promise in activating specific subsets of γδ T cells in vivo in experimental ovarian cancer xenografts [3] and other tumors in a clinical trial [9], leading to significant immune-environment reprogramming in treated tumors. Furthermore, γδ T cells can recognize and target tumor cells that express a wide range of stress-induced ligands or antigens associated with malignancy including, for instance, MICA, MICB, and LETAL/RAET1E [10]. This characteristic makes their anti-tumor activity less dependent on the heterogeneity of the expression of antigens presented through MHC molecules in tumor cells, differentiating them from αβ T cells. However, the impact of the immunosuppressive microenvironment of gynecologic tumors on the anti-tumor effectiveness of γδ T cells remains a complex and poorly understood aspect, including the prioritization of specific subsets for immunotherapeutic modulation. Thus, although γδ T cells show great promise in cancer immunotherapy, extensive research is needed to fully understand their functions and to develop γδ T cell-based superior immunotherapies. In this review, we present an overview of different developments in the field, including planned or possible clinical trials that use γδ T cells to treat gynecologic cancer.

### 1.1. γδ T Cell Subsets: Humans Are Not Mice

Most mouse and human γδ T cell subsets and the butyrophilins that activate some of them, as explained below, do not have clear counterparts as both γδ TCRs and butyrophilins appear to have diverged through evolution. Mouse γδ T cell subsets are usually categorized based on their Vγ chain usage, while human γδ T cell subsets are often characterized according to the expression of Vδ chains [1]. In addition to differences in Vγ and Vδ chain usage, globally, human and mouse γδ T cells also appear to be functionally different. For instance, studies performed in mouse tumor models, including our own work on ovarian cancer [11], identified IL-17- or galectin-1-producing γδ T cells as tumor-promoting, immunosuppressive cell types. In contrast, IL-17-producing γδ T cells are nearly absent in human peripheral blood [12] or peripheral tissues, even under skewing conditions [13], whereas most γδ TILs show strong effector phenotypes in multiple human cancers (manuscript in preparation).

Human γδ T cells are usually categorized depending on the δ chain of their TCR, with three dominant subsets that use either Vδ1, Vδ2, or Vδ3 [8]. These δ chains can be combined with one of the six functional TRGV genes (Vγ2, Vγ3, Vγ4, Vγ5, Vγ8, and Vγ9) [7]. Consequently, different combinations of γ/δ chains determine which stimuli activate the TCR of each subset, although it remains theoretically possible that some conserved ligands could activate multiple TCRs. Accordingly, γδ T cells show tissue-specific localization of oligoclonal subpopulations sharing the same TCR chains. In peripheral blood, for instance, Vγ9Vδ2 T cells account for 60–90% of γδ T cells. Vγ9Vδ2 T cells begin populating the periphery after birth, reach the proportions seen in adulthood in infancy, and show contraction in subjects that are older [14]. This subset of lymphocytes but not other γδ T cell subsets respond to a complex formed by members of the butyrophilin family that is induced by intracellular phosphometabolites. In contrast, the dominant subsets of γδ T cells in organs and solid tumors express either δ1 or δ3 chains, and Vδ3 lymphocytes are barely represented in the blood of most healthy subjects [15,16,17].

Vδ1 T cells mediate antiviral responses [18]. Their frequency varies with ethnicity [14] but Vδ1 T cells undergo clonal expansion shortly after birth, likely in response to viral infections. This results in narrower TCR repertoires and the acquisition of effector phenotypes [19]. Members of the CD1 family—such as the lipid-presenting proteins CD1c and CD1d [20,21], or CD1b [22]—in addition to R-Phycoerythrin [23] all can activate Vδ1 TCRs, at least in vitro. In addition, Vδ1 T cells recognize EphA2 in response to tumor-induced AMPK-dependent metabolic alterations [24]. Vδ3 T cells appear to be functionally similar to Vδ1 T cells but they are abundant in healthy liver [25]. Vγ8Vδ3 T cells have been shown to recognize the metabolite-presenting MR1 protein [26,27]. It is unclear whether Vγ8Vδ1 T cells show similar activity or whether Vδ3 T cells could recognize the same butyrophilin-like molecules that activate subsets of Vδ1 T cells.

### 1.2. γδ T Cells in the Healthy Human Female Reproductive Tract

γδ T cells colonize the mucosa, where they protect against pathogens. Characterizing γδ T cells in the healthy female reproductive tract, however, has been challenging due to significant menstrual fluctuations and age-associated changes [28,29]. Nevertheless, as in other tissues and organs, in the endocervix γδ T cells are predominantly Vδ1^+^, as opposed to the Vγ9Vδ2^+^ γδ T-cell populations predominantly found in peripheral blood [30].

In the mouse endometrium, Kang et al. found enrichment of CD44^high^CD27^high^ γδT cells with attributes of tissue-resident memory differentiation [31]. These cells expressed markers of effector activity and produced high levels of IL-17 upon stimulation, which was attributed to the promotion of the invasion of murine trophocytes. Given the differences in IL-17 production between human and mouse γδ T cells, future studies should clarify the true nature of γδ T cells in the human reproductive tract. Nevertheless, decidual γδT cells in humans have also been associated with the promotion of trophoblast proliferation and invasion, albeit through the production of immunosuppressive cytokines such as IL-10 or TGF-β. Healthy pregnant women show an accumulation of circulating Vδ1^+^ γδ T cells, whereas women with recurrent abortions accumulate Vδ2^+^ circulating cells [32]. Although poorly understood, this bias appears to be required for normal pregnancy. In addition, Hayday and colleagues identified another subset of IFN-γ-producing γδ T cells, which was enriched in young mice and required for protection against *Candida Albicans* [33].

### 1.3. Activation of Cytotoxic and Non-Cytotoxic Functions of γδ T Cells: The Key Role of Butyrophilins

Human γδ T cells can be activated by innate natural killer cell receptors, independently of TCR signaling [30,34]. Thus, activation through DNAM-1, NKp30, NKp44, or, primarily, NKG2D, elicits the cytotoxic activity of human γδ T cells. In addition, γδ T cells can be activated through their TCR. However, the γδ TCR does not respond to classical MHC-peptide structures. Instead, these TCRs are activated by a range of yet incompletely understood “self” molecules that are expressed [35], or change their conformation, in response to cell stress (e.g., CD1 molecules, with or without lipid [36]). γδ T cell activation results in the release of perforin and a variety of granzymes, or killing through ligands that engage death receptors, such as Fas and TRAIL-R. Activation of human γδ T cells also induces the production of effector cytokines such as IFNγ, along with other chemokines and cytokines that have not been properly investigated (e.g., IL-32, lymphotoxin B or granulysin; unpublished observations). In addition, subsets of γδ T cells have been shown to cross-present antigens to CD8^+^ T cells [37] and re-direct antibodies for antibody-dependent cellular cytotoxicity through CD16 [34]. This could be particularly relevant in the context of ovarian or endometrial cancers, the progression of which is heavily dependent on spontaneous production of antibodies in the tumor microenvironment [38,39,40]. Thus, γδ T cells link innate and adaptive immunity through mechanisms that are very different from those of conventional αβ T cells.

**Figure 1:**
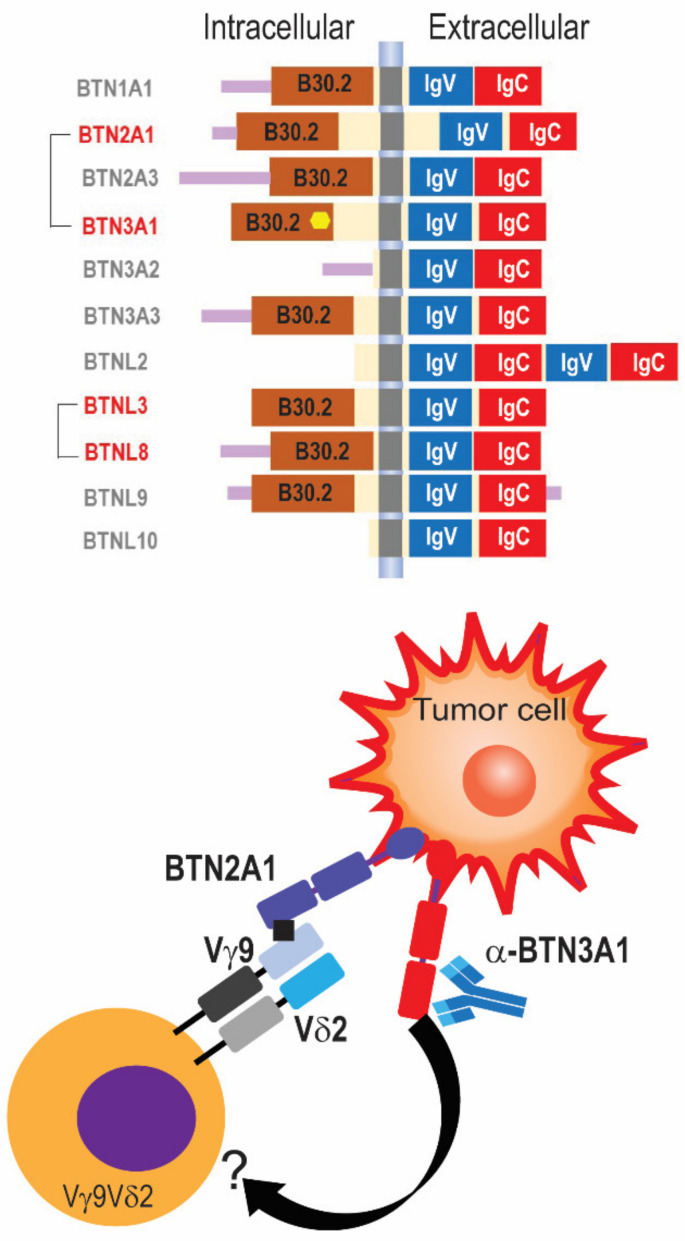
Members of the butyrophlin family of proteins activate different human γδ TCRs. (**Top**) Schematic depiction of members of the family of butyrophilins, including extracellular immunoglobulin domains. Intracellular signaling domains are not present in BTN3A2 and BTNL2. BTNL10 has been recently proposed as a pseudogene. Proteins that cluster together for known γδ TCR activations are shown in red. (**Bottom**) Anti-BTN3A1 agonistic antibodies induce the formation of a protein complex with BTN2A1, which directly binds to the Vγ9 chain of the Vγ9Vδ2 TCR, while BTN3A1 binds to an unknown partner on the γδ T cell surface, eliciting T cell activation.

Among the molecules known to activate different γδ TCRs, butyrophilins and butyrophilin-like molecules have been the subject of intense research in recent years. Most of the 10 functional genes encoding these transmembrane proteins (Figure 1, top) localize to the telomeric end of the MHC complex at Chr6. Polymorphisms in BTN/BTNLs are associated with inflammatory diseases [41,42,43]. Among the members of the butyrophilin family with higher preferential expression in the female reproductive tract, BTNL2 is expressed in the ovaries, whereas BTN2A1 is expressed in the uterus and fallopian tube, according to Genotype-Tissue Expression (GTEx).

Butyrophilins and γδ T cells have diverged between humans and rodents throughout evolution. Investigations on the immunobiology of butyrophilins have been therefore limited by the lack of clear human/mouse counterparts, which precludes relevant studies in KO mouse models. In humans, butyrophilins BTN3A1, BTN3A2, and BTN3A3 share a similar extracellular domain with a CD277 epitope and are among the best-understood members of the family. We and others contributed to demonstrate that the activation of the main subset of γδ T cells in blood (Vγ9Vδ2 T cells) can be elicited through the use of Abs, or phosphometabolites binding to the yuxtamembrane domain of BTN3A1, which promotes a protein complex between this butyrophilin and BTN2A1 and binds to the Vγ9 chain of the Vγ9Vδ2 TCR complex, resulting in the activation of this specific subset of circulating T cells (Figure 1, bottom) [3,44,45,46]. We previously reported that, in their spontaneous conformation, BTN3A butyrophilins inhibit αβ TCR activation by preventing the segregation of N-glycosylated CD45 from the immune synapse, which is required for TCR engagement [3]. Accordingly, CD277 Abs, or zoledronate, which induces the accumulation of BTN3A1-binding isopentenyl pyrophosphate (IPP), restored αβ T-cell effector activity by inducing clustering of BTN3A1 and BTN2A1 that released BTN3A:CD45 engagement [3]. This mechanism can be leveraged to re-direct δ2 TILs against BTN3A1^+^ cancer cells, abrogating malignant progression [3].

Other butyrophilin-like molecules activate different human γδ TCRs. For instance, Vγ4Vδ1 T cells, which primarily reside in the gut, are activated by BTNL3:BTNL8 heterodimers in a “superantigen-like” CDR3-independent manner [16,47]. Whether other “orphan” butyrophilin or butyrophilin-like proteins could bind other Vδ1 or Vδ3 TCRs through the germline-encoded regions of different gamma chains remains to be investigated. Understanding this elusive immunobiology is nevertheless important because firstly, multiple members of this family are expressed in different human cancers, according to TCGA datasets; and secondly, butyrophilin-like molecules expressed in the female genital tract could determine the subsets of γδ T cells that are typically found in the healthy female reproductive tract at different stages of the menstrual cycle.

### 1.4. γδ T Cells in Human Ovarian Cancer

**Figure 2:**
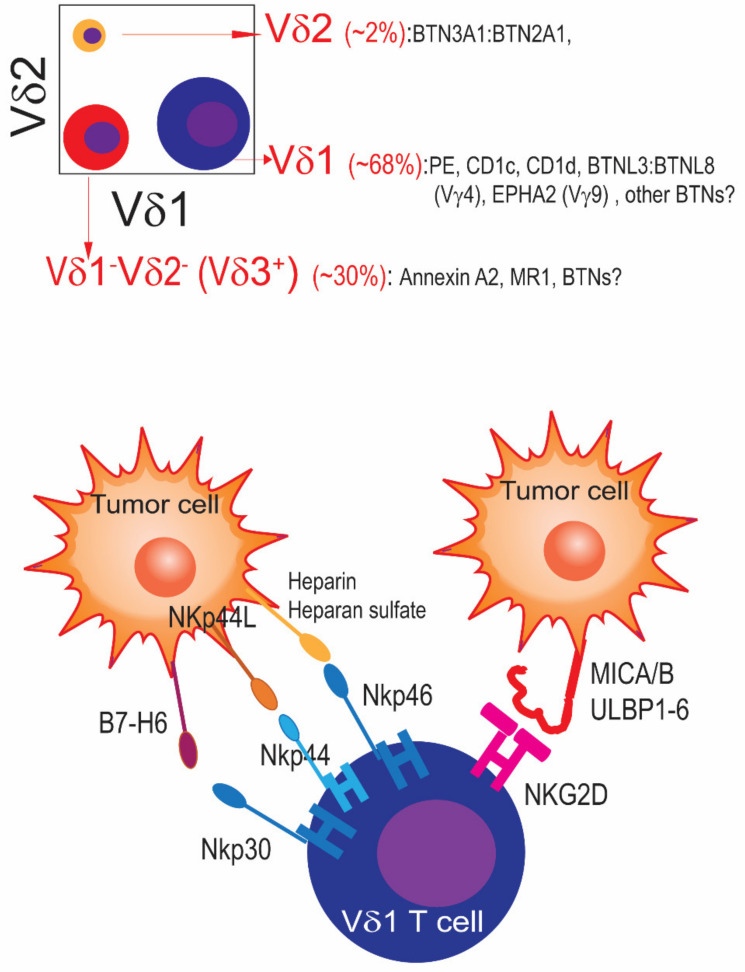
Representative distribution of γδ T cells in human ovarian cancer and known γδ TCR ligands. (**Top**) In contrast to circulated blood, tumor tissues are enriched in γδ T cells expressing Vδ1 or Vδ3 chains, known molecular ligands for each subset are depicted. (**Bottom**) Vδ1 T cells can elicit tumor-cell killing independently to its TCRs through multiple additional mechanisms mediated by natural cytotoxicity receptors such as NKp46, NKp44, NKp30, and NKG2D.

In ovarian cancer, the presence of intra-epithelial CD3^+^ T cells in treatment-naïve tumors has been associated with superior outcomes [48,49]. Our independent studies later showed that γδ T cells, which represent ~6% of total T cells in this disease [3], are a significant component of an effective anti-tumor immune response. Thus, our group reported for the first time the association between the density of infiltration of γδ T cells in treatment-naïve high-grade serous ovarian cancer and overall survival, in addition to responsiveness to BTN3A1-targeted antibodies [3]. Through the analysis of 65 high-grade serous ovarian cancers, our study showed significant enrichment of both the expression of BTN3A1 and the accumulation of γδ T cells in human ovarian cancer compared to tumor-free ovaries or the fallopian tube [3]. γδ T cells represented ~6% of total CD3^+^ TILs on average for nine freshly dissociated ovarian carcinomas, with ranging values in some tumors up to 14% of the total TILs (Figure 2, top). Although we found (BTN3A1-reactive) Vγ9Vδ2 γδ T cells in all specimens (up to 2.5% of TILs), γδ TILs predominantly expressed Vδ1 in seven out of nine of these specimens, whereas populations of Vδ1^−^Vδ2^−^ γδ TILs were dominant in the other two tumors. Both Vδ1 and Vδ3 T cells outnumbered Vγ9Vδ2 T cells in every specimen [3]. Subsequent analyses of Vδ1^−^Vδ2^−^ T cells showed dominant expression of Vδ3 (manuscript in preparation). Therefore, the dominant populations of γδ T cells that spontaneously home to human ovarian cancer are different from the γδ T cells that predominantly circulate through human blood. Tumor homing of γδ T cells could be crucial for selecting suitable cell types and optimal allogeneic cellular therapies to treat solid tumors, such as ovarian carcinomas. Nevertheless, targeting BTN3A1 with novel human antibodies was sufficient to (1) revert the suppression of TCR–antigen engagement with conventional αβ TILs elicited by BTN3A1; and (2) activate Vγ9Vδ2 T cells. Together, αβ and γδ T cells elicited superior control of established ovarian cancer xenografts in response to these BTN3A1 antibodies, in a manner that was superior to PD-1 checkpoint therapy and dependent on the expression of a second butyrophilin (BTN2A1) [3]. Similarly, Foord et al. showed that γδ T cells in ovarian cancer ascites exhibited higher clonality and features of tissue-resident differentiation. In addition, cytokine production by tumor-derived γδ T cells was associated with enlarged overall survival, further supporting the anti-tumor role of γδ TILs. Interestingly, the activity of γδ T cells depended on CD39^+^ conventional T cells, suggesting that CD39 is a possible driver of decreased γδ T cell activity in this disease, and therefore a therapeutic target. It should also be noted that most ovarian cancers express the NKG2D ligands MICA and MICB [50], along with LETAL/RAET1E [10], and could be therefore sensitive to γδ T cell cytotoxic activity independently of TCR activation (Figure 2, bottom).

In independent studies, Chen et al. reported that the percentages of Vδ1 T cells were significantly higher in ovarian cancer than in normal ovaries, whereas chemotaxis assays performed with supernatants generated from ovarian cancer tissues induced the recruitment of γδ T cells [51]. Furthermore, ovarian cancer-derived γδ T cells were able to kill ovarian cancer cells, although with reduced cytotoxic activity. Further supporting the effects of the ovarian cancer microenvironment, γδ T cells incubated with tissue supernatants reduced the proliferation of CD4^+^ T cells ex vivo. Therefore, γδ T cells exert immune pressure against ovarian cancer progression, despite immunosuppressive networks established in the tumor microenvironment.

In terms of γδ T cell-based interventions in preclinical models of ovarian cancer, armored γδ T cells that secrete humanized anti-PD-1 antibodies elicit improved proliferation and enhanced cytotoxicity against ovarian cancer cells, with significantly enlarged survival in xenograft-bearing NSG mice [52].

### 1.5. γδ T Cells in Other Human Ovarian Cancer Gynecologic Cancers and Non-Gynecological Malignancies

In other gynecologic malignancies, γδ T cell infiltration also appears to play a protective role (Table 1). Similar predictive values were independently associated between high TRDV1 (expressed by Vδ1 γδ T cells) levels and improved survival in endometrial cancer [53]. Furthermore, in a recent clinical trial, patients with endometrial carcinosarcoma who did not progress after treatment with cabozantinib in combination with PD-1 blockade showed significantly higher proportions of activated tissue-resident (CD103^+^CD69^+^) ɣδ T cells than progressors [54].

In cervical cancer, decreased γδ T cell numbers are associated with cancer progression [55], suggesting a protective role. In addition, a combination of γδ-T cells and galectin-1 neutralizing antibodies were effective in xenograft models of cervical cancer [62], further supporting the anti-tumor activity of γδ T cells in this disease.

Consistent with all these observations, most studies in other human cancers have also identified γδ T cell infiltration with superior outcomes [7]. This includes lung [4,5], breast [8], hepatocellular [56], renal [6], gastric [57], head and neck [58], and bladder [59] cancers, in addition to melanoma [60]; however, there have been some conflicting reports regarding the role of γδ T cells in tumors such as colorectal cancer, with studies supporting anti-tumor [61] vs. tumor-promoting activities [53] (Table 1).

Notably, in multiple human tumors, PD-1^high^ γδ TILs do not show the genetic and epigenetic signatures associated with quasi-irreversible exhaustion defined by John Wherry and others. Instead, PD-1^+^Vδ1^+^ cells retained effector responses in tumors such as renal [6] and lung cancer [63], as well as melanoma [60], which can effectively respond to PD-1 blockers [60,63]. These results are consistent with our unpublished observations about the phenotype of γδ TILs in human ovarian cancer, which appear to lack the dominant clusters exhibiting overt exhaustion as we found in conventional αβ CD8^+^ TILs with tumor-reactivity attributes [64].

### 1.6. Potential of γδ T Cells in Anti-Cancer Cellular Therapies in Gynecologic Cancers

Although CAR T cells have revolutionized the management of hematological malignancies originating from B cells [65,66,67,68,69,70,71], a combination of immunosuppression, metabolic restrictions, T cell trafficking to tumor beds, persistence, tumor heterogeneity, and the challenge of generating autologous infusion products have prevented the translation of this success to solid tumors so far [72]. The paucity of tumor-specific targets has also led to the testing of CAR T cells re-directed against antigens expressed in healthy vital tissues, such as mesothelin [73] or Folate Receptor alpha (NCT03585764), which presents additional challenges. To overcome this issue, we engineered CAR T cells redirected against ovarian cancer cells through the FSH hormone, the natural ligand of FSHR, expressed in ~60% of ovarian carcinomas of different histological subtypes [74]. This clinical trial is currently enrolling patients at Moffitt Cancer Center (NCT05316129).

γδ T cells could overcome some of the limitations of conventional αβ T cells in cellular therapies against solid tumors due to their resilient effector function at solid tumor beds and the absence of graft-versus-host disease (Table 2). For instance, treating 132 patients with tumors of multiple histological origins with multiple infusions of allogeneic Vγ9Vδ2 T cells, Xu and colleagues identified 8 liver cancer patients and 10 lung cancer patients who showed prolonged survival [75]. Since Vγ9Vδ2 T cells circulate and are dominant in blood, they could be ideal for treating hematological tumors or bone marrow metastases [76]. However, as aforementioned, the γδ T-cell populations that spontaneously home to human gynecologic malignancies (e.g., ovarian cancer [3]) are Vδ1 and, to a lesser extent, Vδ3 lymphocytes. These subsets could be ideal for generating novel allogeneic, “off-the shelf” CAR T cell products that can be used to treat multiple patients. The challenge of this approach has been the expansion of these cells in significant numbers, given their paucity in peripheral blood. Using a proprietary antibody that targets the Vδ1 chain, Adicet has overcome some of these issues in a clinical trial by using CD20 CAR Vδ1 T cells [77]. Using this approach, the company conducted a trial in patients with relapsed or refractory lymphoma. Notably, the persistence of allogeneic Vδ1 T cells at day 28 exceeded that of approved conventional CD19 autologous CAR αβ T therapy. Most importantly, as communicated to ASCO and ASH, there were no occurrences of graft-versus-host disease, paving the way for the use of these subsets of γδ T cells as a safe allogeneic CAR T cell platform in the context of other tumors, including gynecologic malignancies.

We observed that Vδ1 and Vδ3 γδ T cells outnumber Vδ2 T cells in umbilical cord blood. In addition, cord blood Vδ1 T cells show a diverse TCR repertoire, unlike their clonally expanded counterparts in the blood of adult donors [19]. Maintaining this diverse repertoire could be relevant for further activation at tumor beds (e.g., in response to butyrophilins). Expanding Vδ1 and Vδ3 γδ T cells from cord blood is feasible in a scalable manner by using a modified rapid expansion protocol [78]. These cells exhibited an effector phenotype and were enriched in Vδ2^−^ lymphocytes, which were more cytotoxic than their Vδ2 counterparts [78]. Using a different protocol, coupled with αβ T cell depletion, our own group was able to expand from a sample in 2 weeks >10^9^ γδ T cells, which were enriched in Vδ1/Vδ3 subsets by >95% and exhibited an effector phenotype (unpublished observations). Given that γδ TILs appear to be significantly more resilient than their αβ counterparts to exhaustion and functional paralysis at solid tumor beds, allogeneic CAR γδ T cells offer great promise for the treatment of diseases such as ovarian cancer, which develops in a particularly immunosuppressive microenvironment.

### 1.7. Modulating the Phenotype of γδ T Cells in Cancer Patients with Drugs or Antibodies

Antibody-based immunotherapies have revolutionized the management of multiple human cancers. Recently, MMR-deficient patients with endometrial cancers have experienced significant clinical responses upon PD-1 blockade, in combination with chemotherapy [79,80]; however, immune checkpoint inhibitors have so far not produced consistent therapeutic benefits in diseases such as human ovarian cancer [81].

As mentioned above, targeting BTN3A1 with novel human antibodies that promote the formation of a complex of this butyrophilin with BTN2A1 elicited coordinated αβ and Vγ9Vδ2 T cell responses against established ovarian cancer xenografts, including orthotopic tumors [3]. These responses were superior to the immune checkpoint blockade in vivo in tumor-bearing mice. Because ovarian cancer is stubbornly resistant to conventional PD-1 blockers, this study provided a good rationale for testing BTN3A1-modifying, Vγ9Vδ2 T cell-activating antibodies. Although not specifically focused on gynecologic malignancies, ImCheck Therapeutics had previously developed agonistic antibodies with similar activities. The company recently conducted a first-in-human, phase 1/2a clinical study in patients with advanced-stage solid tumors or hematologic malignancies (NCT04243499) [9]. The study included six patients with diverse solid tumors, including a case of ovarian carcinoma. In addition to showing the safety of this approach, all patients showed a decrease in the number of peripheral Vγ9Vδ2 T cells, which exhibited markers of activation. Analysis of pre/post-treatment biopsies from a patient with melanoma showed that treatment elicited increased Vγ9^+^ T cell infiltration compared with the baseline, thus supporting Vγ9Vδ2 T cell activation. Interestingly, the same patient showed that BTN3A1 antibodies elicited greater increases in the accumulation of CD8^+^ T cells producing granzyme B, along with other subsets of γδ T cells [9]. Therefore, while the authors clearly demonstrated that this antibody exerts the activation of Vγ9Vδ2 T cells, preliminary results from this trial so far support the existence of coordinated αβ and γδ T cell responses, as we reported in a preclinical setting.

Aminobisphosphonates, such as zoledronate, inhibit the farnesyl pyrophosphate (FPP) synthase, thereby allowing upstream accumulation of isopentenyl pyrophosphate (IPP), which binds to the yuxtamembrane domain of BTN3A1 triggering the assembly of a protein complex containing BTN2A1 and BTN3A1 that activates the Vγ9Vδ2 TCR [45,82]. In preclinical models, CAR γδ T cells synergize with zoledronate against bone marrow metastases, which has obvious implications for developing future interventions against gynecologic tumors. In addition, anti-tumor immunity could be enhanced in post-menopausal women receiving aminobisphosphonates for osteoporosis; a setting that remains poorly investigated.

## 2. Concluding Remarks

Immuno-oncology has traditionally focused on αβ T cell responses; however, gynecologic tumors, and epithelial cancers in general, are also infiltrated by populations of γδ T cells that exhibit effector phenotypes but not the overt exhaustion of most tumor-reactive αβ T cells [64] or the tumor-promoting features of NK cells in gynecologic malignancies [83]. Because they spontaneously home to peripheral tissues and accumulate at tumor beds, Vδ1 and Vδ3 γδ T cell subsets offer great promise as a platform for chimeric antigen receptors: First, γδ T cells can be used allogeneically, unlike their unmodified αβ T cell counterparts, thus preventing unacceptable waiting times for the generation of autologous infusion products for rapidly deteriorating patients with cancer. In addition, heavily treated patients could not produce a robust infusion product. Second, because γδ T cells could engage tumor cells through both chimeric antigen receptors and a variety of innate receptors that recognize cell stress ligands (e.g., NKG2D), they could be more effective against tumors with high antigenic heterogeneity. Third, γδ T cells persist longer than CD28-costimulated CAR T cells, as was shown in a recent clinical trial, and have been found years after adoptive transfer in CD19 CAR T cell-treated patients [84], which could be relevant for persistent activity against tumor recurrence. Fourth, PD-1 signaling recruits phosphatases that primarily target CD28 and the TCR cascade but innate cytotoxic signals in γδ T cells could be less sensitive to this checkpoint inhibitory signal. Fifth, γδ T cells in tumor beds could re-direct antibodies against tumor cells, and cross-present antigens to conventional lymphocytes.

Not mutually exclusive, there are now clinically available antibodies that safely activate Vδ9Vδ2 T cells in vivo in patients with solid cancers and likely reverse BTN3A-mediated suppression of αβ T cells, re-programming the tumor immune-environment and eliciting significant infiltration of αβ and γδ T cells.

The importance of understanding the role of microbiota in the crosstalk between γδ T cells and tumors should be finally noted. This could provide insights for developing adjuvant immunotherapy with precise regulation of tumor-related microbiota.

Understanding the immunobiology of human γδ T cells and the expression of agonistic butyrophilins in gynecologic cancers could lead to effective, and urgently needed, immunotherapies. This could be particularly valuable for patients with ovarian cancer, who rarely respond to immune checkpoint inhibitors, despite showing features of immune recognition.

**Table 2:** Clinical interventions involving γδ T cells that are currently being tested.

Tumor	Intervention	Trials	References
Multiple advanced-stage	BTN3A1 agonistic Abs	NCT04243499 NCT05307874	[9]
CLL/MM/AML	Vγ9 TCR × CD1d bispecific Abs	NCT04887259	[85]
Lung, liver, AML post-BM transplant	Allogeneic Vγ9Vδ2 T cells	NCT03183219 NCT03183232 NCT05015426	[86]
MRD^+^ AML	Allogeneic Vδ1 T cells	NCT05001451	[87]
Glioblastoma	Temozolomide-resistant γδ T cells	NCT04165941	[88]
Relapsed/refractory solid tumors	NKG2D CAR Vγ9Vδ2 T cells	NCT04107142	[89]
B cell malignancies	Anti-CD20 CAR Vδ1 T cells	NCT04735471	[77]

## Figures and Tables

**Table 1:** Human cancers for which denser γδ T cell infiltration has been associated with better outcomes.

Tumor	Marker/Subset	References
Ovarian carcinoma	Vδ1/Vδ3 γδ T cells	[3]
Endometrial carcinoma	TRD1 (Vδ1 marker)	[53]
Uterine carcinosarcoma	Tissue-resident memory γδ T cells	[54]
Cervical carcinoma	Total γδ T cells	[55]
Non-small-cell lung cancer	Tissue-resident memory Vδ1 γδ T cells	[5]
Breast cancer	Total γδ T cells	[8]
Renal cancer	PD-1^+^Vδ2^neg^ γδ T cells	[6]
Hepatocarcinoma	Tissue-resident memory Vδ2^neg^ γδ T cells	[56]
Gastric cancer	Total γδ T cells	[57]
Head and neck cancer	Total γδ T cells/butyrophilins	[58]
Bladder cancer	Vδ2 γδ T cells	[59]
Melanoma	PD-1^+^Vδ1^+^ γδ T cells	[60]
Colon carcinoma	CD69^+^Vδ1^+^ γδ T cells	[61]
